# Investigating the diversity of scientific methods in high-stakes chemistry examinations in England

**DOI:** 10.1080/09500693.2019.1666216

**Published:** 2019-10-06

**Authors:** Alison Cullinane, Sibel Erduran, Stephen John Wooding

**Affiliations:** a Department of Education, University of Oxford, Oxford, UK; b The Norwegian Centre for Science Education, University of Oslo, Oslo, Norway; c Research and Development, AQA, Manchester, UK

## Abstract

The traditional description of “the scientific method” as a stepwise, linear process of hypothesis testing through experimentation is a myth. Although the teaching and learning of the scientific method have been a curriculum and assessment goal, the notion of the ‘scientific method’ itself has been identified as being problematic. Many researchers have recognised there is no single scientific method. However, there does not seem to be any useful guidelines for how best to deal with the nature of scientific methods in school science, including in high-stakes summative assessment. The article presents the use of a framework to illustrate the diversity of scientific methods that goes beyond the traditional limitations of a scientific method, to provide a more comprehensive and inclusive account, including non-manipulative parameter measurements. The framework not only clarifies the definition of scientific methods but also is adapted as an analytical framework to trace how scientific methods are framed in high-stakes chemistry examination papers from three examination boards in England. Such analyses can potentially point to what is emphasised in chemistry lessons, given how instrumental high-stakes testing is for driving teaching and learning. Results from an empirical investigation of examination questions are presented, highlighting an imbalance in the representation of methods in chemistry tests.

## Introduction

A range of international curriculum policy and research traditions in science education refer to the importance of teaching and learning of the methods of science. For example, the recent reform documents such as the *Next Generation Science Standards* (NGSS lead states, 2013) in the USA refer to statements such as ‘scientific investigations use a variety of methods and tools to make measurements and observations’. In England, the term ‘practical science’ has been widely used in characterising aspects of scientific methods. The Royal Society has used the term ‘practical science’ as ‘ … a shorthand for the full programme of experimental and investigative activities (including fieldwork) conducted as part of science education in schools and colleges’ (House of Lords, 2006, p. 63). The recent policy references follow earlier remarks related to themes such as ‘scientific inquiry’ and ‘scientific practices’. For example, the *National Science Education Standards* made extensive reference to ‘inquiry’ (NRC 1996, p. 31) until recently when ‘scientific practices’ became more prevalent (NGSS lead states, 2013).

One problematic aspect of practical science activities that many international science educators have noted is that despite a succession of reforms in the science curriculum, ‘laboratory activities have engaged students principally in following ritualistic procedures to verify conclusions previously presented by textbooks and teachers’ (Lunetta, Hofstein, & Clough, 2007, p. 396). As Donnelly and colleagues argued, historically school science in England presents a limited set of experiments that do more service to students receiving higher marks than to promote understanding of science or engagement in creative inquiries (Donnelly et al., 1996). According to major reviews of research literature undertaken by Reiss, Abrahams, and Sharpe (2012) and Dillon (2008), there is strong evidence that the assessment regime in England has had a major impact on the amount and variety of practical work that many teachers carry out. There is growing concern that the amount and quality of practical work carried out in schools suffer as a result of the impact of the national tests in science. One of the key conclusions of Dillon’s report was that pupils ‘fail to perceive the conceptual and procedural understandings that were the teachers’ intended goals for the laboratory activities. Students spend too much time following ‘recipes’ and consequently, practising lower level skills.’ (p. 8).

This paper aims to contribute to the expansion and clarification of how scientific methods can be conceptualised in school science for meaningful learning, particularly through the design of summative assessments. Although the teaching and learning of the scientific method have been a curriculum goal (i.e. be it in the context of scientific inquiry, practices or practical science), the notion of the ‘scientific method’ itself has been identified as being problematic. Halwes (2000) noted that the traditional characterisation of the scientific method as a stepwise and linear process of hypothesis testing through experimentation is a myth. Indeed, many researchers have recognised that there is no single scientific method (Lederman, Abd-El-Khalick, Bell, & Schwartz, 2002). Such recognition of the limitations of conventional characterisations of ‘the scientific method’ as hypothesis testing with experiments, however, do not provide any useful guidelines for how best to deal with the notion of ‘the scientific method’ in school science including in the context of assessment (McComas, 2014).

In order to resolve tensions about the definition of the scientific method and its assessment, we begin with a context of the international policy literature on summative assessment of scientific methods covered as part of practical chemistry in high-stakes tests. Subsequently we turn to the research literature on the scientific method and focus on a framework proposed by Brandon (1994) that addresses some of the limitations of traditional conceptualisations of ‘the scientific method’. Brandon illustrates that not all experiments rely on hypothesis testing, and that not all descriptive work is non-manipulative. Indeed, scientific methods rely on a diversity of approaches including parameter measurements. Hence, Brandon’s framework goes beyond the traditional limitations of scientific method as a linear process of hypothesis testing to provide a more comprehensive and inclusive account. We focus on an empirical investigation that utilised Brandon’s Matrix as an analytical framework to trace how scientific methods are framed in high-stakes chemistry examination papers from three of the most influential examination boards in England. Such analyses can potentially point to what is emphasised in chemistry lessons given how influential high-stakes testing is for driving teaching and learning. In other words, teaching often is driven by the content of assessment and teachers teach to the test and hence, reform in assessment can potentially reform what gets taught in the chemistry classroom.

## Summative assessment of practical science

The public examination systems from around the world are complex endeavours utilising a range of qualifications and involving high-stakes assessments of a large number of pupils. For example, in England the Joint Councils for Qualifications (JCQ) reported in 2016 that 5,368,147 GCSE results were issued. Altogether, 15.4 million scripts were marked and approximately 51,000 examiners were employed by exam boards with the majority involved in the marking and moderation. In the current assessment landscape in England, the assessment of practical science skills has been contentious. Science subjects including chemistry no longer include a ‘hands-on’ assessment of practical science skills. Instead, the final examination papers are intended to have items specifically written to indirectly assess students’ knowledge and understanding of practical science (Ofqual, 2015) The Office of Qualifications and Examinations Regulation advocates that at ages 14–16, pupils should ‘develop their ability to evaluate claims based on science through critical analysis of the methodology, evidence and conclusions, both qualitatively and quantitatively’ (Ofqual, 2015, p. 5). Ofqual is a non-ministerial government organisation that regulates qualifications, exams and tests in England, and until May 2016, vocational qualifications in Northern Ireland.

Although England is not unique in its approach to high-stakes tests for students aged 16, such an approach is currently rare. Only a few counties and districts still continue to use high-stakes assessment as a benchmark for achievement of students at age 16. Rather numerous systems nowadays instead focus on the terminal high school exams. Countries that share similar results in the latest PISA science assessments include Singapore, UK, USA, Canada, Australia, France, Ireland and New Zealand (OECD, 2018). However, when the high-stakes examination age is considered to be 16, Australia, Ireland and France are eliminated. As a result, a broad overview of the remaining countries provide some background on how assessment systems deal with the assessment of practical work in science in countries with similar jurisdiction. In Singapore, from 2018 onwards, skills of practical science are assessed using a summative end-of-course assessment (Singapore Examinations and Assessment Board, 2015). In the states of Vermont and New York in the USA, paper-and-pencil tasks are employed. For example, Vermont used an inquiry task on paper which measures the ability to think scientifically. The task requires test-takers to hypothesise, plan and critique investigations, analyse data, and develop explanations. Vermont has announced that future inquiry tasks will be aligned to the NGSS standards (NGSS, 2013).

Education is devolved in Canada with provincial jurisdictions being responsible for their own education provision. In Alberta and Quebec science assessments are high-stakes at age 15/16. The Provincial Achievement Tests (PATs) are sat by students in Alberta at the end of the school year (Hollins & Reiss, 2016). The PATs consist of multiple-choice questions which sometimes assess practical science skills. Epreuves Uniques is the high-stakes examination taken by students in Quebec while in their 4th year of secondary school (15/16 years old). This science curriculum highly focuses on practical science (Creese, Gonzalez, & Isaacs, 2016; Hollins & Reiss, 2016). The mark on the test accounts for 50% of the final score. Students must have a passing mark (normally 60%) to obtain the Diplôme d’Etudes Secondaires (i.e. school exit examinations). Finally, in New Zealand, the National Certificate of Educational Achievement (NCEA) is the official secondary school qualification (NCEA, 2007). NCEA consists of a combination of internal and external assessments. Internal assessments are used to assess skills and knowledge that cannot be tested in an exam, e.g. speeches, research projects and performances, such as those required for practical work in science. Practical activities do not provide students with a complete set of instructions to follow. Instead, students have some freedom to adopt procedures they choose and decide how to record, analyse and report the data collected (Singh, 2014).

The education system in England (i.e. the context of the empirical study presented in the subsequent sections of this paper) underwent a set of reforms beginning in 2010^1^ that resulted in a revised curriculum for many subjects to be taught from 2015 onwards. Importantly these reforms also brought in a new assessment regime beginning with some core subjects in 2017, with the other subjects, including the sciences, following in 2018–2019. As part of these reforms, non-exam assessments such as coursework or controlled assessments were removed from all subjects other than those where a student’s performance, e.g. dance or drama, or the production of physical objects, e.g. art or Design and Technology (D&T), are the most valid expression of their subject skills and competencies. This means that the vast majority of post-reform subjects are now assessed at age 16 (GCSEs) or 18 (A-levels) using only a set of written exam papers. GCSE stands for General Certificate of Secondary Education. It is an academic qualification generally taken in a number of subjects by pupils in secondary education in England, Wales and Northern Ireland. Each GCSE qualification is in a particular subject such as chemistry and stands alone (although a set could also be pursued). Studies for GCSE examinations generally take place over two or three years depending on the subject.

## From cookbooks to mindful exploration of scientific methods

Many practical science activities involve stepwise procedures that are followed in a formulaic fashion. At best, they involve the formulation of a hypothesis and the design and implementation of an experiment. At worst, they are reminiscent of cookbooks where students follow recipes to complete particular experiments. Leonard (1991) provides a description of a cookbook laboratory exercise when he writes

This student [previously described] is the victim of the overly prescriptive laboratory investigation, typical of those used in college introductory science courses. Such laboratory experiences tend to begin with the instructor explaining to the students, often in some detail, what will happen during the exercise in an attempt to make certain that the student will carry out the exercise ‘correctly.’ The student is then left to follow a lengthy and detailed procedure in the laboratory textbook, which will occasionally call for responses such as describing what happens with the apparatus, making a drawing, or answering a specific question in the spaces provided in the manual. The entire procedure is very prescribed, that is, the student is told what to do in a step-by-step fashion for the entire exercise. (p. 84)

The consequence of these cookbook activities is that students can usually complete so-called cookbook labs with no understanding of what they did. An aspect of recipe following in practical work involves hypothesis testing in experiments. Pupils are indoctrinated into a perspective of science as hypothesis testing, with associated concepts of dependent and independent variables taught without much thinking devoted to how different questions might demand other scientific methods.

Although there are many accounts of the scientific method and its use in science teaching and learning (e.g. Halpin & Swab, 1990; McPherson, 2001), these accounts have been limited in taking a comprehensive approach to the diversity of scientific methods. In contrast, a framework proposed by Brandon (1994) provides an overview of scientific methods. Brandon (1994) illustrates that not all experiments rely on hypothesis testing, and that not all descriptive work is non-manipulative. He represents the connections between experiments and observations in terms of a matrix (i.e. two-by-two table) in which the nature of the investigation (experiment/observation) is related to whether it involves manipulation or not, involves hypothesis testing or parameter measures. He represents the connections between experiments and observations in terms of a two-by-two table reproduced here. The nature of the investigation (experiment/observation) is related to whether or not (a) it involves manipulation and (b) hypothesis testing or parameter measure (see Table 1). According to his analysis, one can think in terms of experiment and non-experiments/observations relative to descriptive versus experimental methods.

**Table 1.** Types of scientific methods (reproduced from Brandon, 1994, p. 63).

	Experiment/observation	
	Manipulate	Not Manipulate
Test Hypothesis	Manipulative hypothesis test	Non-manipulative Hypothesis test
Measure Parameter	Manipulative description or measure	Non-manipulative description or measure

The convergence of evidence from different methods can then be used to lead to a broad explanatory structure. It is not one method or one line of experimental or observational evidence that support complex theoretical claims but several lines of evidence need to be synthesised to bring about the level of theoretical rigour that is typically associated with established scientific knowledge. Components of evidence from these different sources drive explanatory frameworks. Erduran and Dagher (2014) identified examples of methods in chemistry (see Table 2). They drew on the work of Scerri who describes how Mendeleev predicted the existence of the element gallium (or eka-aluminum) through a non-manipulative description coupled with quantitative reasoning about atomic weights. Erduran and Dagher's (2014) adaptation of Scerri's example is repeated here to illustrate Brandon's Matrix further:

Mendeleev could interpolate many of the properties of his predicted elements by considering the properties of the elements on each side of the missing element and hypothesizing that the properties of the middle element would be intermediate between its two neighbors. Sometimes he took the average of all flanking elements, one on each side and those above and below the predicted element. This interpolation in two directions was the method he used to calculate the atomic weights of the elements occupying gaps in his table, at least in principle (Scerri, 2007, p. 132).

Scerri states that it was the French chemist Emile Lecoq De Boisbaudran who subsequently ‘worked independently by empirical means, in ignorance of Mendeleev’s prediction, and proceeded to characterise the new element spectroscopically’ (Scerri, 2007, p. 135). De Boisbaudran tested the hypothesis to investigate the new element's existence. He did this by spectral analysis of an ore and isolated gallium through this method. The manipulative aspect of some chemical methods include: (a) Crookes’ study of gases where pressure and voltage were used as variables in spectroscopic study of elements (e.g. Scerri, 2007, p. 251) as an example of manipulative hypothesis testing, and (b) Rutherford’s artificial transmutation of elements through bombardment of nuclei with protons (e.g. Scerri, 2007, p. 253) as an example of manipulative description. All together these methods, along with numerous others, contributed to the collective and eventual depiction of elements.

**Table 2.** Methods related to the Periodicity of Elements (from Erduran and Dagher, 2014)**.**

	Manipulate	Not Manipulate
Test hypothesis	Manipulative hypothesis test *e.g. Crookes’ study of gases*	Non-manipulative hypothesis test *e.g. De Boisbaudran’s discovery of gallium*
Measure parameter	Manipulative description or measure *e.g. Rutherford’s artificial transmutation of elements*	Non-manipulative description or measure *e.g. Mendeleev’s prediction of gallium*

Overall, Brandon’s matrix provides a framework to aid students' understanding of why they do what they do in scientific investigations, and how the convergence of their findings from different methods can be used to explain their results in their investigation. It provides a *hands-on minds-on* approach to scientific methods and helps students see what it is that they are trying to achieve**.** Therefore, in order to go beyond the ‘cookbook’ problem, a systematic change needs to occur, including the teaching and assessment of practical science. Although some other frameworks are effective at presenting an alternative perspective to the traditional depiction of the scientific method, they do not necessarily address the limitations. For example, ‘the scientists’ toolbox’ produced by Wivagg and Allchin (2002) present a characterisation of methods in science through a concise narrative:

Scientists follow hunches, clues, and questions obtained from observations, earlier claims, reading, etc. They explore how to generate relevant information. They consider possible sources of error. They engage others in interpreting evidence. Results usually lead to more questions. Ideas are refined. Some change, some are abandoned.

However, such approaches such as ‘the scientists’ toolbox’ did not offer the same systematic approach as Brandon’s Matrix. For example, Wivagg and Allchin’s structure does not afford an analytical framework with which to categorise examination questions’ depiction of scientific methods. Brandon’s matrix has thus been adapted for methodological use in investigating how examinations in England represent scientific methods in questions.

## Methodology

The section reviews the research questions, data sources and data analysis techniques that drove the empirical study on the chemistry examination papers from three major examination boards in England. As high-stakes assessment often drives what is taught, the study wanted to investigate what practical methods are assessed in high-stakes examinations. The section describes how Brandon’s Matrix was used as an analytical framework. Considering the relevance of Brandon’s categories for methods in chemistry, how do examinations in school chemistry characterise such categories? This is the primary question that guided the analysis of examination questions.

### Research questions

1. What methods underlie the practical chemistry items in high-stakes exam papers from three exam boards in England?
  - What is the frequency of these practical science methods in the exam papers?
  - How does the coverage of practical methods compare across the exam boards?
2. How are the practical science methods marked and what does this indicate about practical methods on these exam papers?

### Data sources

The data sources are examination papers produced by the three main exam boards for general qualifications. For ethical reasons, the names of the exam boards have been anonymised. The exam boards produce tests every year. Due to the current phase of the curriculum reform, the only examination materials available relating to the new science curriculum from these three exam boards were specimen exam papers. These exam papers will be typical of the post-reform question style and content that students should expect as part of their end-of-course written exams. This material is publicly available from each exam board and was created to help teachers and students prepare for the post-reform summer 2018 national examinations. In England, the science subjects have two ability-based tiers: foundation and higher. The higher tier papers were used in this study as the majority of students sit the higher tier papers. Exam papers are made up of two papers. Paper 1 and 2 assess different curricular content and different exam boards label the topic differently, but ultimately, the content is the same. For example, one exam board's Paper 1 examines the first 5 topics from the curriculum (i) the atomic structure and the periodic table; (iii) bonding, structure, and the properties of matter; (iii) quantitative chemistry, (iv) chemical changes; and (v) energy changes. Paper 2 examines (vi) rate and extent of chemical change; (vii) organic chemistry; (viii) chemical analysis, (ix) chemistry of the atmosphere; and (x) using resources and key ideas. Questions in the papers were examined and determined to be are made up of stems and items. The stem usually sets up the context or premise being asked in the following item or items. It is a candidate’s responses to each item that earns marks according to the content and quality.

### Data analysis

This section describes the empirical investigation undertaken on high-stakes chemistry examination papers from England. This study uses Brandon’s Matrix (1994) framework as an analytical tool to investigate the science practical methods that underpin high-stakes chemistry examination papers in England. This section will describe the methodology used to determine the unit of analysis and show some examples to illustrate the classification process. Examples from the different exam boards mentioned above will be provided to illustrate how they were classified for each category. Due to copyright issues exam board material cannot be shown in this publication, therefore items from the exam papers will only be described to illustrate how Brandon’s Matrix was applied to analyse the papers.

### Unit of analysis and reliability

In order to classify the papers, the authors needed to develop a unit of analysis to ensure that the results were reliable and valid. In order to obtain a baseline for the categorisation of the exam papers using Brandon’s Matrix, the authors individually analysed the examination papers using Brandon’s Matrix as the analytical framework. Once completed, the authors compared their results and percentage agreement was obtained for this first round of analysis, which averaged 67.5–87% for the various papers. The authors discussed the disagreements and the unit of analysis was agreed by focusing on the key common criteria. During this consultation phase, negotiation was needed in order to agree on the unit of analysis and as a result some criteria for classifying the questions were then set. Brandon’s Matrix set out main criteria such as (a) the presence of the manipulation of variables (or not) and (b) the presence of hypothesis testing (or not). Also agreed-upon was (c) the inclusion of science investigations outside of the classroom as practical work (e.g. an investigation of chemists in a pharmaceutical company), (d) the inclusion of mathematics type questions, as mathematics is a skill needed in practical science and when a practical question included the use of mathematics it was classed as a non-manipulative parameter measure. (e) There were cases where the stem would present an investigation that required the manipulation of variables, and therefore could be classed as such, however, the item that followed the stem asked the candidate to make an observation about a very particular aspect of the experiment; such an item would therefore be classed as a non-manipulative parameter measurement type question. (f) Items were not classified when the question that followed had no bearing on the practical investigation discussed and were knowledge type questions. An example of instances is illustrated below.

Sometimes the overall question discussed a particular practical investigation which could be easily classified using the criteria set by the authors, however, the item that followed asked a very specific knowledge type question which didn’t fall into any of Brandon’s Matrix categories. E.g. ‘zinc nitrate can be made by reacting zinc oxide with nitric acid, HNO_3_’ which would be classified in Brandon’s categories, but in some instances, a stem like this would follow an item that would ask the candidate to ‘write a balanced symbol equation for this reaction’ which would not fit into Brandon’s categories. Although the stem of the questions relates to a practical investigation they may have carried out in science class, and the reaction of two reactants to form a product (non-manipulative parameter measure), the items that follows asks the candidate to write a balanced equation which did not relate to the practical work directly, and such occurrences were not classified in the analysis.

Using these procedures for categorisation, the authors reviewed their results again. The marking schemes were also consulted to understand what was required to answer the item for full marks, which helped with the agreed-upon unit of analysis and categorisation of the items. Percentage agreement was once again obtained on this second round of analysis and was above 97.5% for all papers. The percent agreement is often used when the number of themes coded are few (Boyatzis, 1998, pp. 153–154). The percentage agreement is obtained using the method put forward by Miles and Huberman (1994). By comparing the results from the authors, a zero was recorded when an item was categorised the same and a one was recorded when there was a disagreement or no match. All the zeros were counted and divided by the number of items for each paper and multiplied by 100 to get the percentage agreement. Miles and Huberman (1994) suggest anything over 80 percent agreement is a good indicator of reasonable reliability. A description is provided below to illustrate how items from the exam papers were categorised for each of the four of Brandon’s Matrix categories. Due to anonymity and the previously outlined copyright issues of the exam boards, images of the questions are not used and are only described.

#### Manipulative parameter measurement

The following section will describe a question that the authors classified as a manipulative parameter measure type question. The topic from the curriculum examined was rate and extent of chemical change. The stem of the question presents a student investigating the rate of reaction between dilute hydrochloric acid and marble chips (calcium carbonate). The student investigates the rate of reaction by using the same mass but different shaped marble chips A and B. In each investigation and they measured the volume of gas given off when the Marble Chip A and B reacts with the acid. Data collected about the volume of gas given off was presented in a graph. This graph had two lines plotted on the graph; Line A and Line B. The volume of the gas/cm^3^ was on the *y*-axis and this was plotted against time on the *x*-axis. This practical is a manipulative parameter measurement type inquiry as it investigated how changing the shape (variable) of the marble chips influenced the outcome. Although the mass remained the same there was manipulation of a variable; the shape or surface area of a marble chips. There was no hypothesis or prediction made before the set-up of this investigation, the student was simply measuring the outcome. If the candidate was asked to predict outcomes based on, e.g. the shape of the chips, then this would have the potential to fall into hypothesis testing. The item that followed the stem of the question asked the candidates to ‘state how the graph shows that line B gives the results for the larger marble chips’, and thus relate directly to understanding the investigation. In order to answer the item, the candidate would need to understand that there was a manipulation of variables and how it would influence the outcome of the investigation. They then would have to identify this in the graph to draw conclusions from the data that are being presented. They were asked to observe an outcome in this case and therefore it fell into manipulative parameter measurement.

#### Manipulative hypothesis testing

An example considered as a manipulative hypothesis testing item is as follows. Like the item above, the question described here also examines the topic of rate and extent of chemical change from the curriculum. The stem of the question outlines how a student is investigating the effect of changing pressure and changing temperature on the reaction; carbon dioxide + hydrogen ⇌ methane + water, for which they also supply the molecular equation CO_2_(g) + 4H_2_(g) ⇌ CH_4_(g) + 2H_2_(l). The exam paper contains a exam paper, is a table showing the percentage yield of methane in the equilibrium mixture produced under different conditions. The temperature (in °C) ranges from 300°C to 1200°C and the pressure (in atmosphere) ranged from 100 to 400. It states that the student predicts that the reaction between carbon dioxide and hydrogen is endothermic and involves a reduction in the volume of gases. The item then asks the candidate to describe and explain whether the student’s predictions are supported by the reaction and results in the table. This item was classified as manipulative hypothesis testing as the candidate had to examine multiple data points produced by changing the variables shown in the table. They then had to understand the prediction that the student made, and how the variables influenced the outcome of the investigation in order to answer the item. The inclusion of a ‘prediction’ demonstrated that there was a hypothesis being used in this investigation.

#### Non-manipulative parameter measurement

The question chosen to describe non-manipulative parameter measurement examined the curricular topic of bonding, structure, and the properties of matter. The stem of the question presents information on rock salt and the method used to separate salt from rock salt by adding it to cold water. There is no manipulation of variables; an outcome is simply measured in the end, in this case the appearance of salt crystals following heating the contents with a Bunsen burner on an evaporating dish. The item asks the candidate to suggest an improvement to the second step to make sure all the salt is dissolved in the water. This was classed as a non-manipulative parameter measurement type question as the candidate needs to answer the question by observation alone and suggest changes. In both these cases, the stem and the item present non-manipulative parameter measurement. There is no strict measurement taking in this investigation, nor was there any manipulation of variables. Therefore, this is classified as a Non-manipulative parameter measurement.

#### Non-manipulative hypothesis testing

The question examined the curriculum topic of chemical analysis. The question presents the elements chlorine, bromine and iodine as part of Group 7 in the periodic table. A table is provided describing the appearances of chlorine, bromine and iodine at room temperature. The candidate is then asked to predict the appearance of astatine, the element below iodine in Group 7. The candidate is required to produce a hypothesis from the information provided about what the chemical and physical properties of such an element would be, and thus performing a type of hypothesis testing. There is no manipulation of variables, but simply an evaluation of what is known and predicting an outcome.

## Results and findings

This section will present the results from the analysis of the exam papers from each exam board and will present the analysis of both Paper 1 and Paper 2 from the higher tier chemistry GSCE exam. The frequencies with which these items are distributed across the Brandon’s Matrix categories will be detailed, along with the marks attributed to these practical items. The latter was carried out to determine if particular Brandon’s Matrix categories were cognitively more demanding than others. The exam boards were anonymised and were labelled A, B and C so not to identify them directly. This is to ensure that no particular favour or criticism is being made to any individual exam board.

### Exam Board A

Table 3 presents the analysis of Paper 1 from Exam Board A. In this case, the total number of items on the exam paper was 47. Of these, 16 items were determined to assess practical science in chemistry. Only two categories from Brandon’s Matrix were represented in this paper, the least representation of all the papers analysed. The distribution of the items relative to Brandon’s Matrix categories were as follows: No items were categorised as manipulative hypothesis testing, seven as manipulative parameter measurement, no items were categorised as non-manipulative hypothesis testing, and nine items were deemed to be non-manipulative parameter measurement.

**Table 3.** Results for Exam Board A, Exam Paper 1.

Exam Board A: Paper 1				
Total items on exam paper: 47 items		Total marks for exam paper: 100 marks		
	MHT	MPM	Non-MHT	Non-MPM
No. of Items	0	7 (15%)	0	9 (19%)
Marks	0	14 (14%)	0	21 (21%)

The analysis of Paper 2 from Exam Board A is presented in Table 4. The total number of items on the exam paper was 46, 19 of which were determined to assess chemistry practical science. The distribution of the items relative to Brandon’s Matrix categories were as follows: 2 items concerned manipulative hypothesis testing, 6 items tested manipulative parameter measurement, 2 items tested non-manipulative hypothesis testing and 9 items tested non-manipulative parameter measurement.

**Table 4.** Results Exam Board A, Exam Paper 2.

Exam Board A: Paper 2				
Total Items on exam paper: 46		Total marks for exam paper: 100 marks		
	MHT	MPM	Non-MHT	Non-MPM
Items	2 (4%)	6 (13%)	2 (4%)	9 (19.5%)
Marks	5 (5%)	19 (19%)	3 (3%)	18 (18%)

### Exam Board B

Table 5 presents the analysis of Paper 1 from Exam Board B. The total number of items on the exam paper were 43, of these, 14 items were determined to assess practical science. The distribution of the items relative to Brandon’s Matrix categories were as follows: No items were categorised as manipulative hypothesis testing, three items were manipulative parameter measurement, one items non-manipulative hypothesis testing and nine items fell into non-manipulative parameter measurement.

**Table 5.** Results Exam Board B, Exam Paper 1.

Exam Board B: Paper 1				
Total Items on exam paper: 43		Total marks for exam paper: 90 marks		
	MHT	MPM	Non-MHT	Non-MPM
Items	0 (0%)	3 (7%)	1 (2%)	9 (21%)
Marks	0 (0%)	11 (12%)	2 (2%)	13 (14%)

The analysis of Exam Board B, Paper 2 is presented in Table 6. The total number of items on the exam paper were 51, eight more items than Paper 1, of which, 22 items were determined to assess practical science. The distribution of the items relative to Brandon’s Matrix categories were as follows: six was on manipulative hypothesis testing, three on manipulative parameter measurement, one on non-manipulative hypothesis testing and 12 non-manipulative parameter measurement.

**Table 6.** Results Exam Board B, Exam Paper 2.

Exam Board B: Paper 2				
Total Items on exam paper: 51		Total marks for exam paper: 90 marks		
	MHT	MPM	Non-MHT	Non-MPM
Items	6 (12%)	3 (6%)	1 (2%)	12 (23%)
Marks	15 (17%)	6 (7%)	2 (2%)	21 (23%)

### Exam Board C

Table 7 presents the analysis of Paper 1 from Exam Board C. The total number of items on the exam paper was 47 items. Of these, 18 items were determined to assess chemistry practical science. The distribution across Brandon’s Matrix categories were as follows: 1 item tested manipulative hypothesis testing, two items tested manipulative parameter measurement, 1 tested non-manipulative hypothesis testing and 14 items tested non-manipulative parameter measurement.

**Table 7.** Results for Exam Board C, Exam Paper 1.

Exam Board C: Paper 1				
Total number of items: 47		Total marks: 100 marks		
	MHT	MPM	Non-MHT	Non-MPM
Items	1(2%)	2(4%)	1(2%)	14 (30%)
Marks	2 (2%)	4(4%)	3(3%)	24(24%)

**Table 8.** Results for Exam board C, Exam Paper 2.

Exam Board C: Paper 2				
Total number of items: 40		Total marks: 100 marks		
	MHT	MPM	Non-MHT	Non-MPM
Items	1(2.5%)	5(12.5%)	3(7.5%)	11(27.5%)
Marks	6 (6%)	15(15%)	7(7%)	27(27%)

The analysis of Paper 2 from Exam Board C is presented in Table 8. The total number of items on the exam paper were 40, 7 fewer items than Paper 1, of these, 18 items were determined to assess chemistry practical science. The distribution of the items relative to Brandon Matrix categories were as follows: 1 was on manipulative hypothesis testing, 2 on manipulative parameter measurement, 1 on non-manipulative hypothesis testing and 14 non-manipulative parameter measurement.

### Overall trends

**Figure 1.**
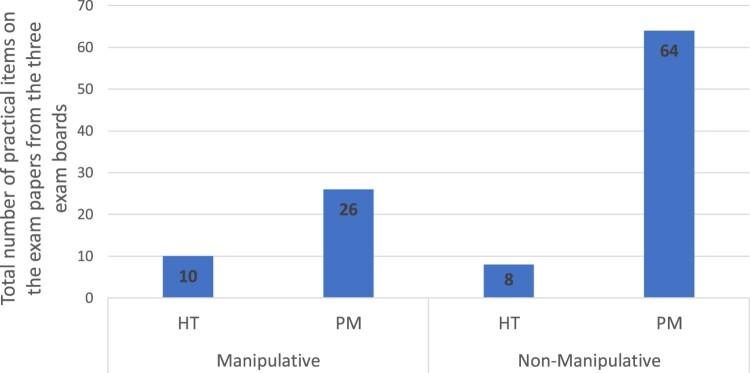
Total practical items on the exam papers from the three exam boards.

Figure 1 presents all the practical items across the three exam boards and shows the overall trends of the Brandon’s Matrix categories. It clearly shows the frequency of categories on the exam papers and illustrates how the different categories compare in their frequency to one another. The distribution of the items relative to Brandon’s Matrix categories were as follows: 10 were manipulative hypothesis testing, 26 on manipulative parameter measurement, 8 on non-manipulative hypothesis testing and 64 non-manipulative parameter measurement.

**Figure 2.**
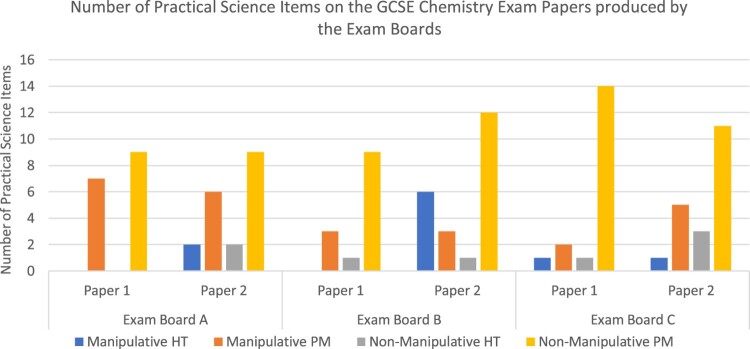
Distribution of items and marks in Exam Papers 1 and 2 across Brandon’s Matrix.

Figure 2 presents the distribution of the items across the exam paper from the different exam boards. The items and the marks are shown to compare how the type of activity are weighted on the exam script. Some of the categories are weighted more than others. This is particularly seen with the manipulative parameter measure category. This often yielded a higher percentage of marks than the percentage of items on the exam paper. The opposite was evident for the non-manipulative parameter measure items. These questions often yielded a higher percentage of items but received less percentage marks. For example, Table 5 (Results Exam Board B, Exam Paper 1) shows that the manipulative parameter measure category made up 7% of the items but received 12% of the marks. On this same table non-manipulative parameter measure items made up 21% of the items but received 14% of the marks. This is perhaps an indication that items that examine manipulative parameter measure assess higher cognitive levels than items related to non-manipulative parameter measure (Erduran et al., 2019, Cullinane & Liston, 2016). Figure 2 compares the items between the exam boards. It shows that the items on the exam papers were comparable to each other. All three exam boards followed similar trends. Paper 1 from both Exam Board A & B do not have manipulative hypothesis testing questions.

The results indicate that for both Paper 1 and Paper 2, non-manipulative parameter measurement was the method assessed at the highest frequency. In both papers, manipulative hypothesis testing was the category with the lowest frequency of items. Furthermore, the mark allocation was the highest in both papers in the non-manipulative parameter measurement category. However, Paper 2 had a higher percentage of items and marks dedicated to manipulative parameter measurement as compared to Paper 1. The trends in the distribution of the categories illustrate a relatively uniform distribution within each paper’s items and allocated marks, although the relative distribution was different across the two papers. Paper 2 had higher percentage of manipulative parameter measurement in both items and the marks as compared to those from Paper 1.

Overall, the pattern suggests consistency between the items allocated to each category and the marks allocated to them across the exam boards. Also consistent was the trend of more marks being allocated to manipulative type questions, even though the relative frequency for the items were lower. In other words, there was relatively more items dedicated to non-manipulative parameter measurement as compared to manipulative parameter measurement. The relative distribution of marks was not consistent suggesting that more marks were dedicated to manipulative parameter measurement as compared to the number of items covered in the exam. Proving, perhaps, an assumption that manipulative parameter measurement is considered to demand a higher cognitive ability thus deserving more marks (e.g. Cullinane and Liston, 2016). Adding to this assumption was the observation that most of the non-manipulative parameter measurement questions appeared in the beginning of the exam papers and therefore usually received only one or two marks. The manipulative parameter measurement questions usually appeared more towards the middle and end of the exam papers and could receive up to 6 marks per item. Perhaps, an indication these practical methods require higher cognitive demands to understand and answer on the exam script, and maybe even perform. (e.g. Cullinane & Liston, 2016).

## Conclusions and implications

The introduction of this article discussed how the characterisation of the scientific method displayed in textbooks and school classrooms around the world is a myth. (Halwes, 2000, 1998, McComas, 1998, 2014, Wivagg & Allchin, 2002). It presents science as a hypothetico-deductive model that reduces science to a series of steps. The model is not an accurate reflection of how science works, especially when you try to apply it to studies in evolution, climate change and astronomy, to name but a few examples. However, the narrow view of science continues to be portrayed to those studying science, at all levels. Although the literature shows the various myths of ‘the scientific method’, no other frameworks have been put forward to offer a systematic alternative, as offered by Brandon’s Matrix (1994). Brandon's Matrix is unique as it illustrates the wealth of techniques and approaches being used in science. It is also unique, as it offers flexibility in its utility as an analytical tool, as our study shows. It also allows us to rethink the goals for science instruction so that explicit reference can be made to these various methods in science curricula.

The empirical component of the paper illustrated Brandon’s Matrix as an analytical tool and showed what methods underlie the practical chemistry items in high-stakes examination papers. The paper illustrates the need to design assessments that place emphasis on a more balanced representation of methods in chemistry. The finding in this paper illustrate how manipulative parameter measurement dominated the exam papers and how manipulative hypothesis testing type questions were present in a limited capacity. This was contrary to initial belief that manipulative hypothesis testing would be dominant, as this is often presented as ‘the scientific method’ in many science classrooms around the world. This inconsistency between the well-established ‘the scientific method’ and the presentation of science methods in high-stakes examination is a recipe for confusion, as well as leading to cookbook style procedures in the science classroom It is not difficult to understand why students are confused by methodological procedures in science (Leonard, 1991). This study therefore shows there is a disconnect with what is being presented as methods in science, the methods they are performing to draw conclusions from investigations and the methods of practical science that they are tested on.

Summative assessment often drives what is taught, so much so, that teachers are routinely influenced by methods that appear in summative assessments (Abrahams, Reiss, & Sharpe, 2013; Cullinane and Liston, 2016). Therefore, future assessment can potentially shift the balance between the assessment of practical skills and the cognitive reasoning skills necessary for science. The article not only raises questions about the design of examination items to assess student understanding of practical science, but also about the design of curricular content that aims to advance high-level thinking and reasoning skills about methods used in science. Student learning of practical science should be evaluated in order to go beyond mindless following of recipe-style science investigations or following prepared instruction (Cramman et al., 2019). Ensuring a balanced approach to the representation of methods in assessment items will likely lead to a more diverse range of methods being used in teaching, thus improving students’ understanding of how different methods work in chemistry to produce knowledge. Future research can further investigate how scientific methods are positioned in school science teaching and learning, and in particular how students are prepared for being tested about practical science in national high-stake assessments. Considering that the breadth and scope of practical work undertaken by students is limited in England (SCORE, 2008), it is vital that future efforts to ensure that national examinations can be put to good use by promoting meaningful teaching and learning of practical science.

## Note

1 A full list of all documentation referring to the reforms is available at the time of writing from https://www.gov.uk/government/collections/gcse-as-and-a-level-reforms
